# Comparative Genomic Analysis of *Citrobacter* and Key Genes Essential for the Pathogenicity of *Citrobacter koseri*

**DOI:** 10.3389/fmicb.2019.02774

**Published:** 2019-12-06

**Authors:** Chao Yuan, Zhiqiu Yin, Junyue Wang, Chengqian Qian, Yi Wei, Si Zhang, Lingyan Jiang, Bin Liu

**Affiliations:** ^1^Key Laboratory of Molecular Microbiology and Technology, Ministry of Education, TEDA College, Nankai University, Tianjin, China; ^2^TEDA Institute of Biological Sciences and Biotechnology, Nankai University, Tianjin, China; ^3^Tianjin Research Center for Functional Genomics and Biochips, TEDA College, Nankai University, Tianjin, China; ^4^Tianjin Key Laboratory of Microbial Functional Genomics, TEDA College, Nankai University, Tianjin, China; ^5^College of Life Sciences, Nankai University, Tianjin, China

**Keywords:** *Citrobacter koseri*, whole genome sequence, comparative genomic analysis, pathogenicity, high-pathogenicity island

## Abstract

*Citrobacter* species are opportunistic bacterial pathogens that have been implicated in both nosocomial and community-acquired infections. Among the genus *Citrobacter*, *Citrobacter koseri* is often isolated from clinical material, and has been known to cause meningitis and brain abscess in neonates and immunocompromised individuals. The virulence determinants of *Citrobacter*, however, remain largely unknown. Based on traditional methods, the genus *Citrobacter* has been divided into 11 species, but this has been problematic. Here, we determined an improved, detailed, and more accurate phylogeny of the genus *Citrobacter* based on whole genome sequence (WGS) data from 129 *Citrobacter* genomes, 31 of which were sequenced in this study. A maximum likelihood (ML) phylogeny constructed with core genome single-nucleotide polymorphisms (SNPs) classified all *Citrobacter* isolates into 11 distinct groups, with all *C. koseri* strains clustering into a single group. For comprehensive and systematic comparative genomic analyses, we investigated the distribution of virulence factors, resistance genes, and macromolecular secretion systems among the *Citrobacter* genus. Moreover, combined with group-specific genes analysis, we identified a key gene cluster for iron transport, which is present in the *C. koseri* group, but absent in other the groups, suggesting that the high-pathogenicity island (HPI) cluster may be important for the pathogenicity of *C. koseri*. Animal experiments showed that loss of the HPI cluster significantly decreased *C. koseri* virulence in mice and rat. Further, we provide evidence to explain why *Citrobacter freundii* is less susceptible than *C. koseri* to several antibiotics *in silico*. Overall, our data reveal novel virulence clusters specific to the predominantly pathogenic *C. koseri* strains, which form the basis for elucidating the virulence mechanisms underlying these important pathogens.

## Introduction

The *Citrobacter* genus belongs to the Enterobacteriaceae family, which is a distinct group of aerobic, Gram-negative, non-spore-forming, rod-shaped bacteria that typically utilize citrate as their primary carbon source (Janda et al., 1994; Sakazaki, 1997). *Citrobacter* species are commonly found in water, soil, and food, and occasionally colonize the gastrointestinal tract of animals and humans (Arens and Verbist, 1997). Although *Citrobacter* strains colonizing the human gastrointestinal tract are generally considered to have low virulence (Pepperell et al., 2002), they may cause a wide range of diseases in the urinary tract, respiratory tract, bone, peritoneum, endocardium, meninges, intestines, bloodstream, and central nervous system, particularly in infants, young children, and immunocompromised adults (Altmann et al., 1976; Fincher et al., 1990).

The majority of infections are associated with *Citrobacter koseri* (Hodges et al., 1978; Lipsky et al., 1980; Lavigne et al., 2007; Mohanty et al., 2007). *Citrobacter* infections usually supervene upon debilitated, hospitalized patients, with multiple comorbidities (Gross et al., 1973; Williams et al., 1984; Kline and Kaplan, 1987; Mohanty et al., 2007; Samonis et al., 2009). Urinary tract infections caused by *Citrobacter* account for approximately half of all infections, although there are no reports of statistically significant associations between infection sites and *Citrobacter* species, except for *C. koseri*, which exhibits a remarkable degree of tropism for the brain (Gross et al., 1973; Ribeiro et al., 1976; Curless, 1980; Williams et al., 1984; Kline and Kaplan, 1987; Doran, 1999).

Based on traditional methods, the *Citrobacter* genus has been divided into 11 species (Brenner et al., 1993, 1999). Phylogenetic relationships based on 16S ribosomal RNA (rRNA) sequences and multilocus sequence analysis (MLSA) based on partial sequences of *rpoB*, *pyrG*, *fusA*, and *leuS* have been used to discriminate *Citrobacter* species (Warren et al., 2000; Clermont et al., 2015). However, gene sequence variation provides limited resolution to discriminate between closely related members of the *Enterobacteriaceae* family (Naum et al., 2008). Whole genome sequence (WGS) is now being employed for routine surveillance and for the detection of possible outbreaks in many countries due to lower cost, simpler protocols, and reduced time investments (Kaas et al., 2012; Dallman et al., 2015), which provides the opportunity to resolve bacterial strains at the single-nucleotide resolution needed for identifying cases linked to a common infection source (Ashton et al., 2016) and for clustering isolates into higher taxonomic groups.

Our past research has established experimental and *in silico* serotyping systems for *Citrobacter* based on specific genes in O-antigen biosynthesis gene clusters (Qian et al., 2018). In this work, we aimed to generate a fine-scaled, more accurate phylogeny and population structure based on whole genome data of *Citrobacter* species. Moreover, we performed an affiliation of *Citrobacter* species and comparative genomic analysis to gain a better understanding of virulence and resistance gene distribution in *Citrobacter*. Furthermore, we identified key genes contributing to *C. koseri* virulence. We also found explored why *Citrobacter freundii* is considered less susceptible than *C. koseri* to several antibiotics *in silico*.

## Materials and Methods

### Bacterial Strains, Plasmids, and DNA Extraction

Bacterial strains, plasmids, and primers used in this study are listed in Supplementary Table S1. The 31 *Citrobacter* strains sequenced in this study were obtained from the Polish Collection of Microorganisms (PCM) at the Hirszfeld Institute of Immunology and Experimental Therapy, Polish Academy of Sciences (Wrocław, Poland). All strains were stored at −80°C in Luria-Bertani (LB) broth supplemented with 20% (v/v) glycerol and were cultured at 37°C in LB broth. When necessary, chloramphenicol was used at a final concentrations of 15 μg/ml. A Bacteria Extraction Kit (CWBIO Co., Ltd., China) was used for DNA extractions from each strain according to the manufacturer’s instructions. Mutant strains were generated using a λRed Recombinase System (Kirill and Barry, 2000), and all strains were verified via PCR amplification and sequencing. To generate ΔHPI (high-pathogenicity island) mutant strains, HPI cluster gene fragments were replaced by the chloramphenicol acetyltransferase cassette.

### Genome Sequencing and Assembly

The whole genome of strain TBCP-5362 was sequenced using a PacBio RS II (Pacific Biosciences, Menlo Park, CA, United States), with a depth of approximately 100-fold coverage. The reads produced with the PacBio RS II were *de novo* assembled using MaSuRCA (Tallon et al., 2014; Kuang et al., 2015). The other 30 strains were sequenced using Illumina Paired-End sequencing technology (Illumina, Little Chesterford, Essex, United Kingdom), with a depth of 90–100-fold coverage. A library for Illumina Paired-End sequencing was prepared from 5 μg DNA using a Paired-End DNA Sample Prep Kit (Pe-102-1001, Illumina Inc., Cambridge, United Kingdom). Libraries prepared using Nextera technology and paired end reads of either 100 bp (Illumina HiSeq 2000). DNA was fragmented by nebulization for 6 min at a pressure of 32 psi. For end-repair and phosphorylation, sheared DNA was purified using the QIAquick Nucleotide Removal Kit (Qiagen, Crawley, United Kingdom). The end-repaired DNA was A-tailed and adaptors were ligated according to the manufacturer’s instructions. *De novo* assembly was performed using Velvet Optimiser v2.2 (Zerbino and Birney, 2008). Genome sequence annotation was conducted using the National Center for Biotechnology Information (NCBI) Prokaryotic Genome Annotation Pipeline^1^. In addition, 98 publicly available *Citrobacter* genomes were obtained from NCBI GenBank.

### Identification of Gene Orthologous Groups

OrthoFinder (Emms and Kelly, 2015) was used to determine orthologous families of the pan-genome with default parameter (for BLASTp: outfmt = 6, *e*-vaule = 0.001; for MCL: *I* = 1.5). All protein sequences were compared using a BLASTp “all-against-all” search with an *E*-value cutoff of < 1e−3. The single-copy core gene, pan gene, and core genome families were extracted from the OrthoFinder output file. Nucleotide sequences of single-copy core genes were extracted according to protein ID.

### Phylogenetic Analysis

According to the identification of gene orthologous clusters, a total of 1450 single-copy orthologous core genes were found to be shared per genome. To determine the single-nucleotide polymorphisms (SNPs), the nucleotide sequences of single-copy core genes identified by core genome phylogenetic analysis were aligned using MAFFT (Katoh and Standley, 2013). The SNPs were integrated according to the arrangement of the single-copy genes in the *C. koseri* TBCP-5362 genomes. The phylogeny of SNPs was inferred using the maximum likelihood (ML) algorithm in PhyML (with the GTR model of nucleotide substitution and c-distributed rates among sites). MEGA7 and FigTree v1.4.3^2^ were employed to construct the trees. In consideration of homologous recombination caused by horizontal gene transfer occurring in bacterial populations which can confound phylogenetic analysis, we identified and removed putative recombination regions in the set of SNPs of single-copy core genes using CloneFrameML (Didelot and Wilson, 2015). Neighbor Net (NNet) splits graph based on uncorrected p-distances were constructed and visualized with SplitsTree 4 (Huson and Bryant, 2006).

### Whole-Genome Nucleotide Identity

Average nucleotide identity (ANI) and tetramer usage pattern were calculated for the 129 *Citrobacter* genome datasets using JSpecies 1.2.1 (Richter and Rosselló-Móra, 2009) and Gegenees v3.0 (Agren et al., 2012), using default parameters. The results were visualized using the pheatmap R package.

### Core and Pan-Genome Analysis

Core and pan-genome analyses were separately performed using the 129 *Citrobacter* genomes. The regression analysis for the core gene cluster curve was performed using a weighted least square regression by fitting the power law *n* = κexp(*m* ×*N*) + Θ to means (Bottacini et al., 2010). *N* is the number of genomes, *n* is the number of core gene clusters, Θ is a constant value representing the predicted minimum number of core genes, and κ and *m* are parameters. According to Heap’s law pan-genome model (Tettelin et al., 2008), the total number of gene clusters is shown for increasing number of genomes (*N*). The curve was a least squares fit of the power law *n* = :*N*^γ^ to averages. An exponent γ > 0 indicates an open pan-genome species.

### Species-Specific Core Genome Comparison

To examine the pan-genome in greater detail, we constructed a cluster map of the gene families across all 129 genomes using the heatmap clustering command from the pheatmap R package (Figure 3B). We excluded the core gene families and low frequency gene families that are shared by <10 strains. We termed the results as the “Group 8-specific core genome” (Supplementary Table S5), which represents the set of gene families that are shared across all strains of a species.

### Gene Functional Category

We analyzed the functional category of the core gene families and the Group 8-specific core genome using different database (COG/GO/KEGG) and compute the numbers of proteins for each corresponding COG/GO/KEGG term. For different database, we use Cluster of Orthologous Group (COG) assignment (Galperin et al., 2015), InterProScan 5 (Jones et al., 2014), and WEGO 2.0 (Ye et al., 2006), BlastKOALA^3^, using default parameters, respectively. We determined the main biological function of differential proteins using function enrichment analysis and results visualized by GraphPad Prism 7.0.

### Identification of Macromolecular Secretion Systems and Gene Island (GI)

The detection and visualization of macromolecular systems in the *Citrobacter* genus were performed using the programs MacSyFinder (Abby et al., 2014) and TXSScan (Abby and Rocha, 2017), using default parameters^4^. Furthermore, the type VI secretion system (T6SS) was predicted as described previously (Boyer et al., 2009) and combined with the results obtained using SecReT6 (Li et al., 2015), using default parameters. Genomic Island (GI) was detected using IslandViewer 4, using default parameters^5^ (Bertelli et al., 2017).

### Calculation of Codon Usage and GC Content

We used CodonW software^6^ to compute statistical parameters of nucleotide composition of gene clusters and genomes.

### Identification of Virulence Genes and Resistance Genes

We made use of the large-scale BLAST score ratio (LS-BSR) tool (Sahl et al., 2014), which was run against the Virulence Factors Database (Liu et al., 2019) and the Comprehensive Antibiotic Resistance Database (Jia et al., 2017). We removed virulence factors of previously studied macromolecular secretion systems and the lipopolysaccharide (LPS). Heatmaps showing the distribution of virulence factors and resistance genes were generated using the pheatmap R package.

### Animal Infection

Laboratory animals, BALB/c mice (18 days old) and Sprague Dawley (SD) rats (2 days old), were purchased from Beijing Vital River Laboratory Animal Technology Co., Ltd. (Beijing, China). All mice were maintained in a specific pathogen-free environment. 18-day-old mice received bacteria in 100 μl phosphate-buffered saline (PBS) via the tail vein (Zhu et al., 2010). Two-day-old rat pups were anesthetized with isoflurane and inoculated with bacteria in 100 μl PBS via intraperitoneal injection. Blood and cerebral spinal fluid (CSF) samples were collected from anesthetized rat pups at the indicated time points. Blood and CSF samples were aseptically collected via intracardiac and cisterna magna punctures, respectively, as previously described (Kim et al., 1992). CSF (5 μl) and blood samples (50 μl) were inoculated into Luria broth and agar plates with antibiotic selection. Rats were then euthanized, and whole brains were removed.

### Ethics Statement

The Institutional Animal Care Committee at Nankai University (Tianjin, China) approved all animal research procedures. Every effort was made to minimize animal suffering and to reduce the number of animals used.

## Results and Discussion

### Genomic Features, Phylogenetic Analysis, and Species Taxonomy

**FIGURE 1:**
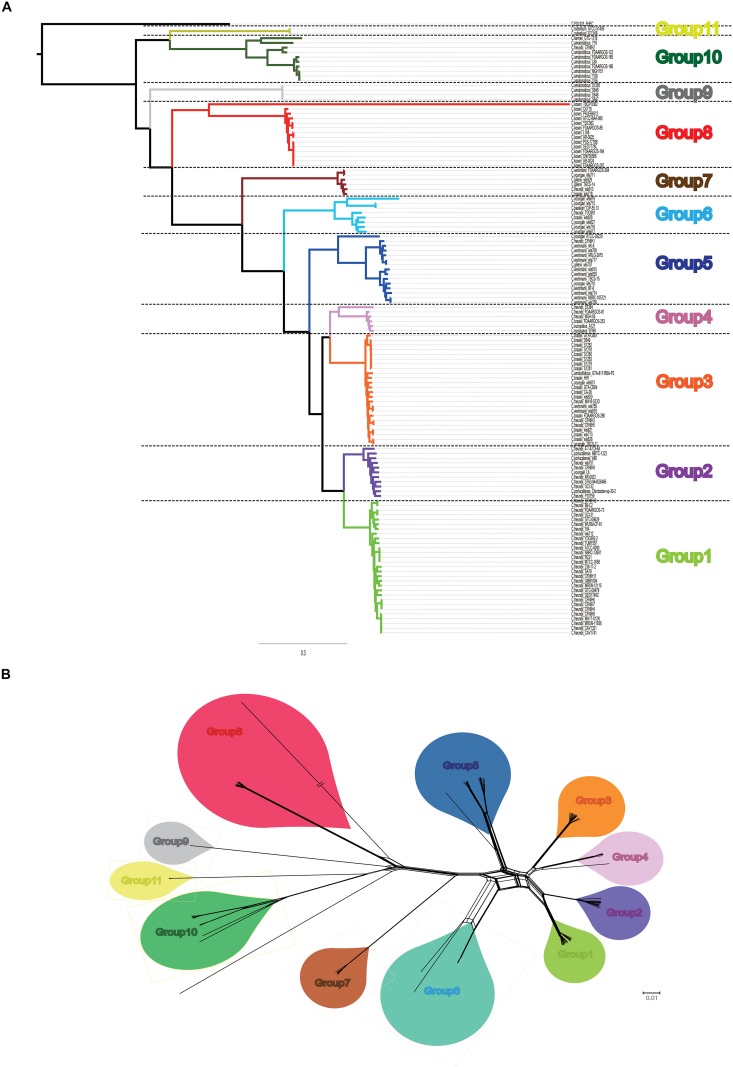
Core genome-based phylogeny. **(A)** Maximum likelihood tree was constructed using PhyML based on 1450 single-copy core genes shared by 129 *Citrobacter* strains. Different group are shown in different colors. **(B)** Neighbor Net (NNet) splits graphs based on uncorrected p-distances inferred from 1450 single-copy core genes shared by 129 Citrobacter strains. NNet splits graphs were constructed and visualized with SplitsTree4. Different group are shown in different colors, which was corresponding to our phylogenetic tree.

**FIGURE 2:**
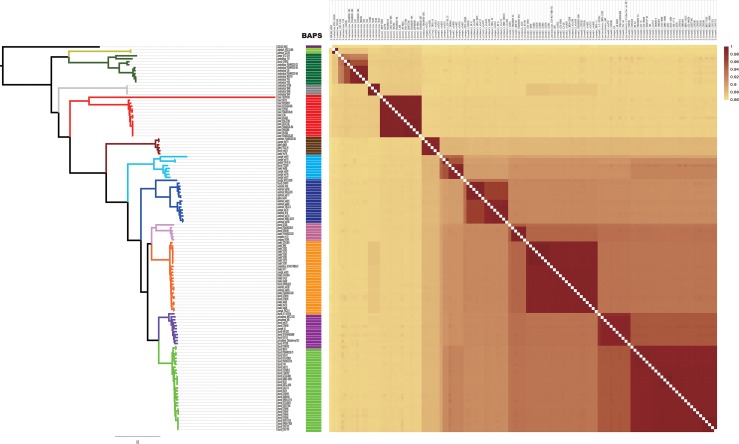
Heatmap chart generated from distances calculated based on the ANI values of 129 *Citrobacter* strains. The colors in the heatmap represent pairwise average nucleotide identity (ANI) values, with a gradient from yellow (low identity) to dark red (high identity).

A summary of the features of each of the 129 *Citrobacter* genomes is shown in Supplementary Table S1. The GC content of the genomes ranged from 51 to 56%. The genome sizes varied from 4.0 to 5.0 Mb, with the number of coding sequences ranging from 4277 to 5904. In order to assess the phylogenetic relationship of *Citrobacter* species, a ML phylogeny tree was constructed using the concatenated nucleotide sequence of 1450 core genes from 31 newly sequenced and 98 publicly available *Citrobacter* strains. The core genome tree generated a reliable delineation of phylogenetic relationships across the *Citrobacter* genus. According to the core genome tree, the 129 strains were divided into 11 lineages (Figure 1A). To further explore the genomic similarities among strains, we supplemented phylogenies with genetic population structure analysis using Bayesian analysis of population structure (BAPS) (Cheng et al., 2013) and the ANI value was calculated to estimate the genetic distance between strains at the genomic level (Richter and Rosselló-Móra, 2009). ANI value was calculated using two software JSpecies 1.2.1 (Figure 2) and Gegenees v3.0 (Supplementary Figure S1). Population structure analysis assigned the *Citrobacter* genus into 11 groups (Groups 1–11) (Figure 1), which corresponded to our core genome tree. To further group *Citrobacter* in a phylogenetic context, NNet splits graph was generated (Figure 1B). The splits in the network serve the same purpose as the familiar branches in a phylogenetic tree, that is, they separate the sequences into 11 groups, one either side of the branch or split. We found that Groups 8 and 11 were specific to *C. koseri* and *Citrobacter rodentium*, respectively. *Citrobacter amalonaticus* was distributed in Groups 9 and 10. Though Group 1 only had *C. freundii*, this species was distributed in all groups except Groups 8 and 11. The distribution of other species was not group-specific. Because of its global presence, intraspecific variation and diverse behavior in strains led to ambiguity in *Citrobacter* classification. Hence, WGS has been helpful for allowing us to investigate the genomes and classify them accordingly. Based on whole genome data of *Citrobacter*, we constructed a high-resolution, more accurate phylogeny and population structure, which represented a significant improvement over that based on the conventional classification system.

### Characterizing the Core and Pan-Genomes

**FIGURE 3:**
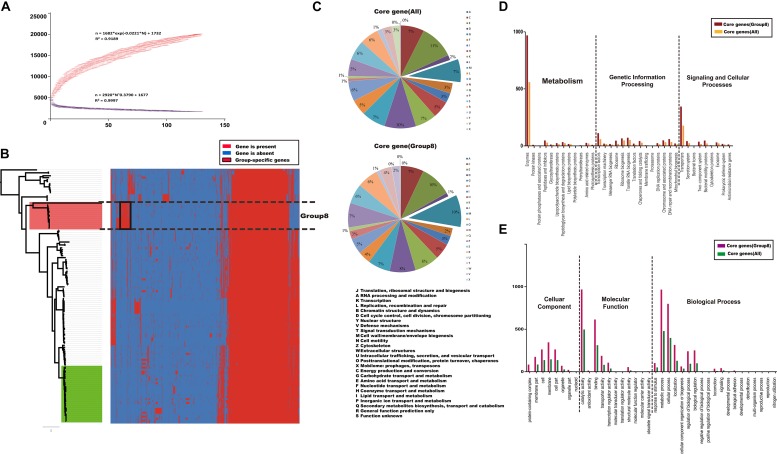
Core and pan-genome analysis of *Citrobacter* strains. **(A)** The *Citrobacter* core and pan-genome plotted were constructed for 129 genome sequences of *Citrobacter*. **(B)** Cluster map of the accessory genome of *Citrobacter*. The gene families that are unique to a species and conserved across most of strains in that species are framed in black. **(C–E)** Distribution of functional catalogs of core genes of all *Citrobacter* strains and Group 8 only after COG/GO/KEGG annotation, respectively.

To assess genetic diversity, we constructed core and pan-genome plotted of the *Citrobacter* genus (Figure 3A). From the *Citrobacter* pan-genome, 20114 gene families were identified across 129 genomes, of which 1450 constituted the core genome. We further used the Cluster of Orthologous Group (COG) assignments to identify the functional categories of the core gene families of all *Citrobacter* species and those specifically of Group 8 (*C. koseri*). The core gene families were unevenly distributed across the functional categories (Figure 3C). There were several obvious differences between all groups and Group 8 in the numbers of genes belonging to the same COG category, such as transport and metabolism of carbohydrates (category G); translation, and ribosomal structure and biogenesis (category J); and inorganic ion transport and metabolism (category P). It is noteworthy that most of the core gene differences were related to transport and metabolism. Bacterial signal transduction systems are responsible for sensing environmental cues and adjusting cellular behavior and/or metabolism in response to such cues. Besides, we also analyzed the functional category of the core genes shared by 129 *Citrobacter* and the Group 8-specific core genes using two other different database (GO/KEGG) and compute the numbers of proteins for each corresponding GO/KEGG term (Figures 3D,E). Results indicate that 8 gene functional categories were enriched in both core gene families and the Group 8-specific core genome based on GO functional annotations. Including “cell,” “cell part,” “membrane,” “binding,” “catalytic,” “localization,” “cellular process,” and “metabolism process” (Figure 3E). For KEGG annotations, two gene functional categories were enriched in both core gene families and the Group 8-specific core genome including “Enzymes” and “Transporters” (Figure 3D). Thus, we hypothesized that genes related to transport and metabolism might provide a competitive advantage to *C. koseri* infecting humans, which may lead to unique pathogenicity.

### Comparative Genomics Analysis of Virulence Genes in *Citrobacter*

**FIGURE 4:**
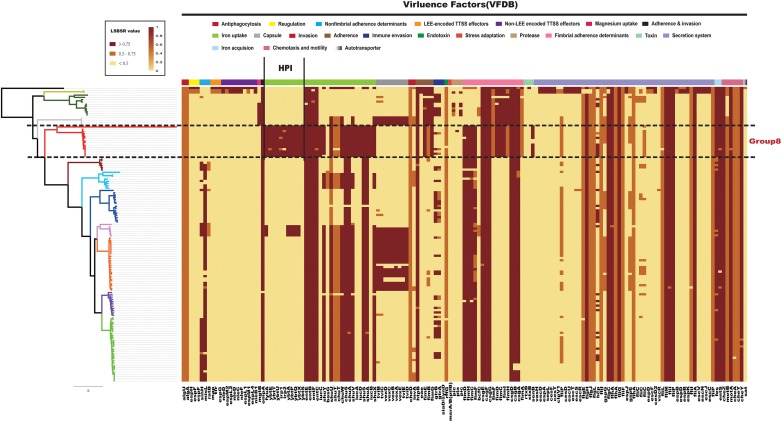
Distribution heatmap of virulence factors across *Citrobacter* strains. Color coding for virulence factors corresponds to the score ratio recorded for each genome when screened with the reference Virulence Factors Database. For a full outline of sources, see Supplementary Table S2. In the current presentation, we removed the virulence factors of previously studied macromolecular secretion systems and O-antigen/LPS/Capsule.

Previously research made use of infant mouse models to study the pathogenicity of *C. koseri* (Soriano et al., 1991). Some *in vitro* studies have shown that *C. koseri* is able to invade and replicate inside human U937 macrophages and human brain microvascular endothelial cells (Badger et al., 1999; Townsend et al., 2003). It has also been demonstrated that *fliP* influences the uptake of *C*. *koseri* by macrophages, as well as cytokine expression and brain abscess formation in neonatal rats (Townsend et al., 2006). However, there is limited information on the virulence genes of *C. koseri*. To identify key pathogenicity genes of *C. koseri*, we investigated the distribution of virulence genes in *Citrobacter*. All 129 *Citrobacter* genomes were locally aligned against the Virulence Factors Database (Liu et al., 2019) to detect virulence genes (Supplementary Table S2). We found that the major virulence factors identified in all strains were associated with flagellar apparatus biosynthesis (*ompA*, *csg* fimbriae, and the *che* operon) and iron uptake (*chu*, *fep*, and *ent*). Specifically, Group 8, which consisted of *C. koseri* only, contained a virulence gene cluster (*VFG000358–VFG000368*) (Figure 4), which formed a complete HPI found in highly pathogenic strains of the genus *Yersinia*. This gene cluster encodes a siderophore-mediated iron uptake system that is required for full virulence in *Yersinia* (Carniel, 1999). Thus, we speculated that this genomic feature may contribute to the pathogenic effects on the central nervous system (CNS) during *C. koseri* infection, which is rarely observed for other related species. Group 3, which mainly consisted of *C. freundii* and *Citrobacter braakii*, contained the genes *VFG000423–VFG000430*, which encode the Vi capsule polysaccharide of *Salmonella enterica* subsp. *Enterica* serovar Typhi str. CT18 (*Salmonella* Typhi, for short). The Vi capsule enables *Salmonella* Typhi to avoid host defenses and is important in enhancing infectivity and virulence (Joan and John, 1984; Hone et al., 1988; Arya, 2002). These genes may provide bacteria higher potential for pathogenicity and adaption within humans. Group 11, which consisted of *C. rodentium*, contained genes that encoded well characterized type III secretion system (T3SS) components (Schauer et al., 1995; Luperchio et al., 2000; Vallance et al., 2003).

**FIGURE 5:**
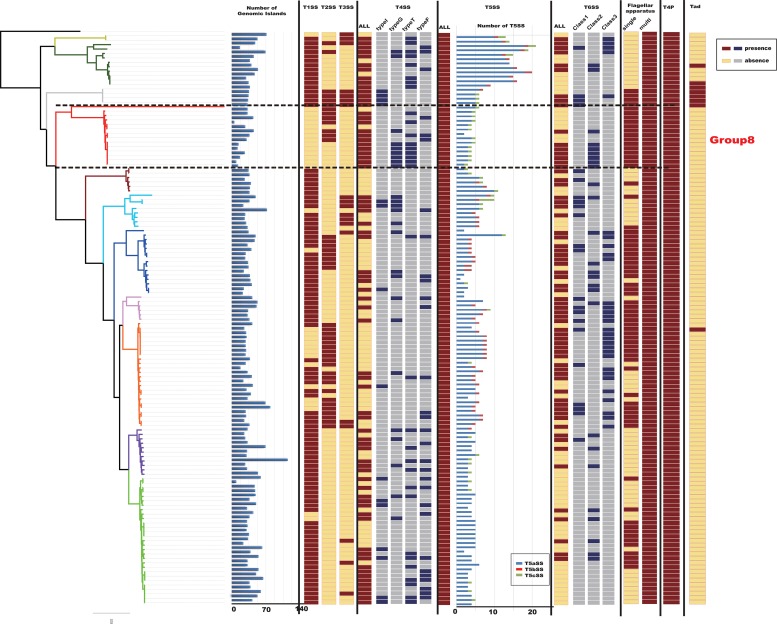
Macromolecular secretion system and genomic island (GI) distribution in *Citrobacter* strains. Dark red colors and blue boxes represent the presence of a macromolecular system within a genome, while gray colors and yellow boxes indicate the absence of a macromolecular system.

Genomic islands play an important role in the evolution, adaptation, and diversification of bacterial genomes, carrying genes that encode proteins with diverse functions (Juhas et al., 2009). Distinct properties of GIs allow bacterial organisms to evolve and adapt to different environments, it is possible to understand why they spread rapidly (Juhas et al., 2007). This adaptation process is among the most important factors in generating diversity and facilitating the propagation of genes in bacteria, as the organism receives an already prepared and improved set of genes, increasing its chances of adaptation (Wilson, 2012). We detected GIs in the 129 *Citrobacter* genomes using IslandViewer 4 (Figure 5). Results indicated that *Citrobacter* strains harbor diverse and heterogeneous GIs and the distribution of GIs-number of 129 *Citrobacter* is irregular. The average-GIs-number of Citrobacter was 42, which may provide *Citrobacter* with diversity functions like ability to adapt to more environment and virulence.

Protein secretion is essential for bacteria to interact with their surrounding environments (Abby and Rocha, 2017). In particular, pathogenic bacteria secrete many virulence factors (Raymond et al., 2013). To further analyze the virulence genes of *Citrobacter*, we investigated the occurrences of macromolecular secretion systems in the 129 *Citrobacter* genomes using the MacSyFinder (Figure 5). We found that Flagellar apparatus, Tad pilus, type IV pilus, and type V secretion system (T5SS) are restricted to the *Citrobacter* genus, while type II secretion system (T2SS), type III secretion system (T3SS), type IV secretion system (T4SS), type V secretion system (T5SS), and type VI secretion system (T6SS) are shared by some strains within the genus. However, the strains in Group 8 did not have type I secretion system (T1SS), type III secretion system (T3SS), one kind of type IV secretion system (T4SS), one kind of type V secretion system (T5SS), and Tad pilus, which might be related to higher success in colonizing the human environment.

**FIGURE 6:**
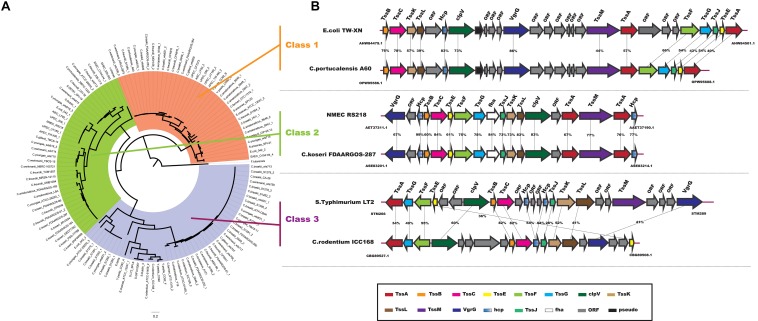
Model of T6SS in *Citrobacter* strains. **(A)** Phylogenetic tree of select T6SS gene clusters. The figure was made using the sequences of the *TssF* core component homologs. **(B)** Genes encoding the T6SS-1, T6SS-2, and T6SS-3 in the indicated *Citrobacter* strains are shown schematically. Homologous genes are colored similarly (see box below).

In particular, the T6SS is a multiprotein machine, widespread in Gram-negative proteobacteria, and especially common in Gamma-proteobacteria (Coulthurst, 2013; Zoued et al., 2014). Previous work showed that *Escherchia coli* T6SSs can be categorized into three distinct phylogenetic groups: T6SS-1 to T6SS-3 (Russell et al., 2014). In our study, using the same method in *E. coli*, we performed a phylogenetic analysis of the *Citrobacter* genus and other classified strains including *Salmonella* LT2, NMEC RS218, EAEC 042, APEC TW-XN, *Pseudomonas aeruginosa, Vibro cholerae*, *Edwardsiella tarda*, and *Francisella tularensis* to classify the T6SSs into three classes, as shown in Figure 6A. The phylogenetic tree suggests that T6SS gene cluster in *Citrobacter* genus might be acquired by horizontal gene transfer (HGT). The functions associated with the three classes are quite different. T6SS-1 proteins are involved in biofilm formation; T6SS-2 proteins are commonly involved in colonization, survival, or invasion (often of human hosts); and T6SS-3 proteins include antibacterial effectors. We found that only T6SS-2 genes were present in Group 8. Nearly half of the strains in Group 8 have T6SS-2 genes, which may be related to the unique pathogenicity of these strains in their host environments. This idea should be further explored in future studies. A model of the three *Citrobacter* T6SS gene cluster classes is shown in Figure 6B.

### Resistance Genes Distribution of *Citrobacter*

**FIGURE 7:**
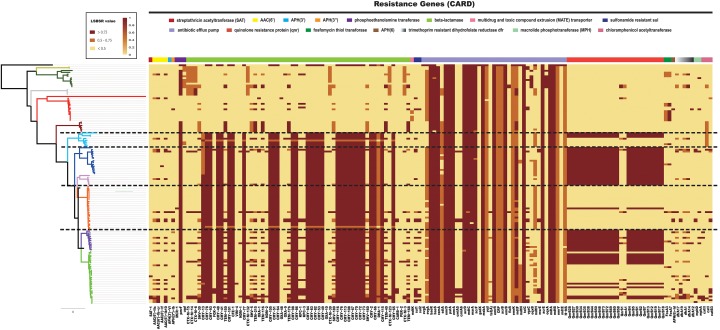
Distribution heatmap of resistance genes across *Citrobacter* strains. Color coding for resistance genes corresponds to the score ratio recorded for each genome when screened with the reference The Comprehensive Antibiotic Resistance Database. For a full outline of sources, see Supplementary Table S3.

Previous research has shown that resistance to β-lactams is common among *Citrobacter* and is mediated through production of β-lactamases. *C. freundii* is considered inherently resistant to many antimicrobial agents including amoxicillin, amoxicillin-clavulanate, ampicillin, ampicillin/sulbactam, first- and second-generation cephalosporins, and cephamycins (Patel, 2001). It has been suggested that *C. freundii* is less susceptible than *C. koseri* to several antibiotics (Samonis et al., 1987). To explore this possibility, all 129 *Citrobacter* genomes were locally aligned against the ResFinder database (Zankari et al., 2012) to detect resistance genes (Supplementary Table S3). We identified a number of resistance genes within the *Citrobacter* genus (Figure 7). We found that all species had genes which encoded different types of antibiotic efflux pumps, including resistance-nodulation-cell division (RND) types, major-facilitator superfamily (MFS) types, ATP-binding cassette (ABC) types, which confer resistant to aminoglycoside antibiotics, aminocoumarin antibiotics, fluoroquinolone, lincosamide antibiotics, cephalosporin, cephamycin, and penam (Supplementary Table S4). Genes encoding β-lactamase were distributed in all *Citrobacter* strains except those in Group 8, which rarely have such genes. Strains in Groups 9 and 10 primarily contained CTX-M β-lactamase genes for resistance to cephalosporin, while strains in Groups 1–7 consistently contained CMY β-lactamase genes. In particular, genes encoding the quinolone resistance protein Qnr were mainly distributed in Groups 1, 2, 4, 5, and 6. This finding is in agreement with a previous study, which suggested that *Citrobacter* spp. may be the origin of *qnrB* genes, a hypothesis based on species distribution (>60% in *Citrobacter* spp.) (Kehrenberg et al., 2008; Jacoby et al., 2011; Saga et al., 2013) and that the *qnrB* gene is prevalent in *C. freundii* strains isolated from human clinical specimens (Park et al., 2007). Other work supports *Citrobacter* as the origin of *qnrB* as this gene is distributed in strains including *C. freundii, C. braakii, Citrobacter youngae, and Citrobacter werkmanii* (Ribeiro et al., 2015). Importantly, strains of Group 8 had less antibiotic-resistant genes than other those in the other groups, especially Groups 1–6, which contained *C. freundii, C. braakii, C. youngae, and C. werkmanii.* Our research showed that *C. koseri* had less resistance genes than other species, which may explain why *C. koseri* is considered more susceptible to several antibiotics. Furthermore, we provided a comprehensive comparative genomics analysis on the distribution of resistance genes in *Citrobacter*, which establishes a foundation for the clinical treatment of *Citrobacter* in the future.

### Group-Specific Genes

**FIGURE 8:**
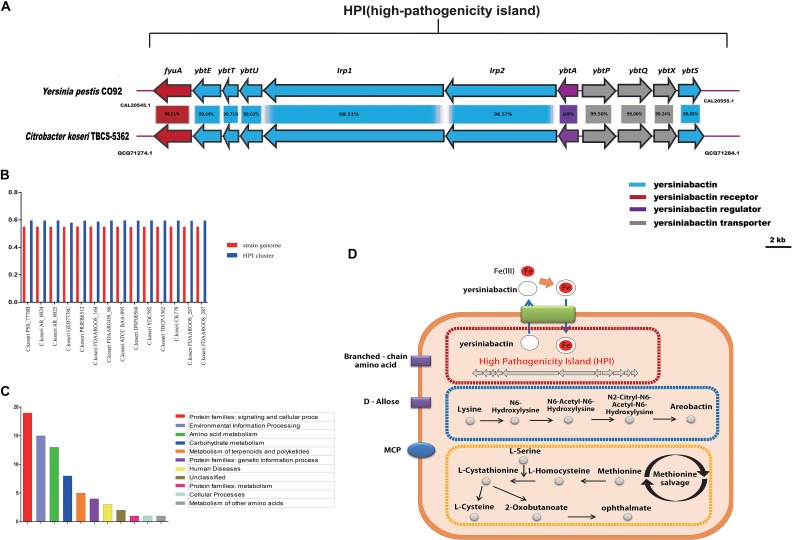
Group 8-specific core genome. **(A)** Model of HPI in *Citrobacter koseri* TBCP-5362. The percentage of protein identity for each set of homologous genes is shown. Homologous genes are colored similarly (see box below). **(B)** GC content of HPI in strains of Group 8. **(C)** Functional enrichment of Group 8-specific core genome after KEGG annotation. **(D)** Sketch map of the Group 8-specific core genome after KEGG annotation.

The group-specific gene analysis revealed underlying profiles of gene families that are conserved among strains within a group, and some of which are unique to a particular group. As such, Group 8-specific genes may provide information not only about virulence of *C. koseri*, but also about the metabolic features of the host environment. To identity group-specific gene families, we constructed an accessory genome by subtracting the core genome and low frequency genes (existence < 10) from the pan-genome. For Group 8, a total of 285 gene families were identified as the group-specific core genome (Supplementary Table S5). Based on KEGG annotation, the functional categories “Protein families: signaling and cellular process,” “Environment information processing,” and “Amino acid metabolism” were enriched in the Group 8-specific core genome (Figure 8C). These group-specific genes may be related to inherent differences in pathogenicity between *C. koseri* and strains of the other groups. Through systemic analysis of these genes, four complete pathway modules, including three ABC transporters and one MCP (methyl-accepting chemotaxis protein), were present in the Group 8-specific core genome. Moreover, we found group-specific genes related to HPI clusters, aerobactin biosynthetic clusters, methionine salvage-related clusters, ABC transporters for D-allose, ABC transporters for branched-chain amino acids, and MCP genes (Figure 8D).

### The HPI Clusters Is Essential for *C. koseri* Pathogenicity

Based on the results of the comparative genomics analysis, we focused on HPI clusters, models of which are shown in Figure 8A. We also examined the GC content of each of the two gene clusters and the three host genomes. Our results showed that the gene clusters displayed apparent deviations in GC content (Figure 8B), which suggests that they may have been acquired by HGT events. Furthermore, we assessed the putative HPI homologs using BLASTp searches in the NCBI non-redundant protein database. We found that the well characterized HPI in *Yersinia pestis* CO92 was closely related to the HPI cluster in *C. koseri* TBCP-5362, and that these gene clusters are present in similar genomic locations and encode proteins with more than 95% sequence identity. Previous studies have shown that HPIs are common in highly pathogenic *Yersinia* strains and that they are required for full virulence (Gehring et al., 1998; Carniel, 1999; Perry et al., 1999). Thus, we speculated that these gene clusters might play a vital role in allowing *C. koseri* to survive in humans and may explain why *C. koseri* exhibits a remarkable degree of tropism for the brain.

**FIGURE 9:**
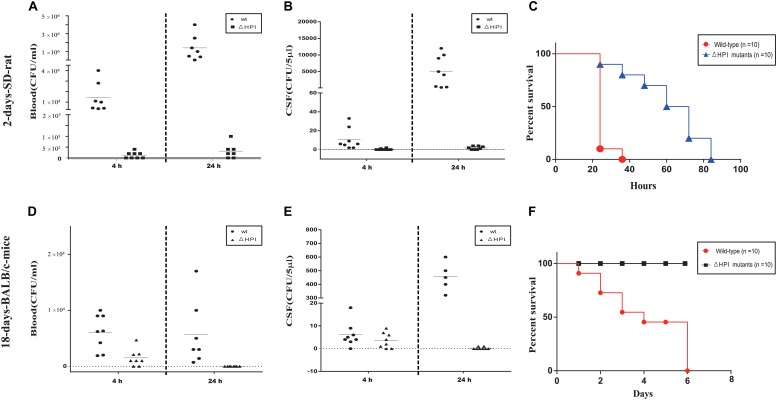
Lack of HPI-cluster decreases *C. koseri* virulence in host. **(A)** Bacterial counts recovered from blood of 2-day-old SD rats. **(B)** Bacterial counts recovered from CSF of 2-day-old SD rats. **(D)** Bacterial counts recovered from blood of 18-day-old BALB/c mice. **(E)** Bacterial counts recovered from CSF of 18-day-old BALB/c mice. Data reflect three independent experiments. Bars represent mean CFUs of all mice, with *P* values determined by the Mann–Whitney *U* test (^∗∗^*P* < 0.01). **(C,F)** Survival plots of 2-day-old SD rats and 18-day-old BALB/c mice after inoculation with wild type or ΔHPI mutant bacteria (∼1 × 10^6^ CFUs for 2-day-old SD rats and ∼2 × 10^7^ CFUs for 18-day-old BALB/c mice). Data presented are the combination of three independent experiments, ^∗∗∗^*P* < 0.001 by log-rank curve comparison test.

To test our hypothesis, we generated a mutant strain that lacks all HPI clusters (ΔHPI:chlR) in *C. koseri* TBCP-5362. As previously described, two animal models were used in our study: 2-day-old SD rats and 18-day-old BALB/c mice (Soriano et al., 1991; Liu et al., 2019). Three groups of each animal model were inoculated with wild-type or ΔHPI mutants of *C. koseri* TBCP-5362 (∼5 × 10^5^ colony forming units (CFUs) for 2-day-old SD rats and ∼1 × 10^7^ CFUs for 18-day-old BALB/c mice). Blood and cerebral spinal fluid (CSF) were collected analyzed. In 18-day-old mice colonized with ΔHPI mutants, we found that the amount of bacteria in CSF was significantly lower than in mice inoculated with wild type bacteria at 24 h post-infection, while in blood, the bacteria concentrations were very similar (Figures 9D,E). Likewise, in 2-day-old SD rats, although colonization with wild type bacteria resulted in increases in both blood and CSF, the higher level of bacteria in CSF (nearly a 500-fold increase) was much greater than what was observed in blood (a nearly 100-fold increase) (Figures 9A,B). For the ΔHPI mutant, colonization was significantly decreased in both blood and CSF in both animal models after 24 h. Surprisingly, in 18-day-old mice, our results suggest that ΔHPI mutants lost the ability to replicate in brain.

In a separate set of experiments, two groups of each animal model were inoculated with wild-type or ΔHPI mutants of *C. koseri* TBCP-5362 (∼1 × 10^6^ CFUs for 2-day-old SD rats and ∼2 × 10^7^ CFUs for 18-day-old BALB/c mice) and survival was monitored. For 2-day-old SD rats, animals infected with wild type started to die by 24 h and all died within 36 h. In contrast, animals infected with ΔHPI mutants started to die by 24 h and all died within 84 h. Similarly, for 18-day-old BALB/c mice, animals infected with wild type started to die by 24 h and all died within 144 h, while animals infected with ΔHPI mutants did not die within the monitored time. Survival curves identified a significant difference between wild type groups and mutants groups (log-rank test, *P* < 0.01, Figures 9C,F).

Overall, our results indicated that deletion of the HPI cluster severely attenuated *C. koseri* virulence *in vivo* and ΔHPI mutants lost the ability to replicate in brain, which suggests that the HPI cluster is a novel virulence cluster that plays a key role in *C. koseri* pathogenicity.

## Conclusion

The genome sequences of 31 *Citrobacter* strains generated in this study are important for the broader goal of using WGS data for comparative studies. In this study, phylogenetic and population structure analyses based on a core genome, in combination with whole genome ANI profiling, provide a clear distinction of *Citrobacter* species into 11 groups.

Our comparative analysis showed differences in macromolecular secretion system profiles among *Citrobacter*. In particular, for Group 8 (the *C. koseri-*specific group), we identified several genes vital for pathogenicity and which may contribute to the high degree of tropism for the brain. These include HPI genes, an aerobactin biosynthetic gene cluster, a methionine-salvage related gene cluster, ABC transporters for D-allose, ABC transporters for branched-chain amino acid, and MCP. We also demonstrated through *in vivo* animal studies that the HPI cluster is essential for *C. koseri* pathogenicity. On the other hand, our research showed that *C. koseri* had less resistance genes than other species, which may explain why *C. koseri* is more susceptible to antibiotics.

## Data Availability Statement

The datasets generated for this study can be accessed from the https://card.mcmaster.ca/, http://www.mgc.ac.cn/VFs/main.htm, NCBI, GenBank: GCA_000732965.1, GCA_000835925.1, GCA_000027085.1, GCA_000764735.1, GCA_000981805.1, GCA_002880615.1, GCA_001559075.1, GCA_001558935.1, GCA_000731055.1, GCA_001471655.1, GCA_002151695.1, GCA_001276125.1, GCA_001276105.1, GCA_002918935.1, GCA_002919495.1, GCA_002918555.1, GCA_002918535.1, GCA_005406305.1, GCA_003226155.1, GCA_000939835.1, GCA_000018045.1, GCF_002863965.1, GCA_000783445.1, GCA_003184045.1, GCA_002947035.1, GCA_001546325.1, GCA_001546305.1, GCA_001471775.1, GCA_001552875.1, GCA_002947675.1, GCA_002208985.3, GCA_002386385.1, GCA_005281055.1, GCA_005280825.1, GCA_005280805.1, GCA_005281195.1, GCA_005280945.1, GCA_005281345.1, GCA_005281015.1, GCA_000826205.1, GCF_002863945.1, GCA_005280855.1, GCA_005280875.1, GCA_005280975.1, GCA_005281175.1, GCA_000155975.1, GCA_000648515.1, GCA_002114305.1, GCA_005281255.1, GCA_002185305.2, GCA_005280955.1, GCA_005281125.1, GCA_005281355.1, GCA_005280845.1, GCA_005280815.1, GCA_005281115.1, GCF_002025225.1, GCA_005281025.1, GCA_000759755.1, GCA_005281305.1, GCA_002918505.1, GCA_000783995.1, GCA_000692115.1, GCA_002073755.2, GCA_900080005.1, GCA_900079995.1, GCA_000786265.1, GCA_002918575.1, GCA_002919425.1, GCA_002918495.1, GCA_002918455.1, GCA_002918465.1, GCA_002919455.1, GCA_002919485.1, GCA_000972645.1, GCA_002923765.1, GCA_005281215.1, GCA_000786275.1, GCA_002939255.1, GCA_005281325.1, GCF_003114935.1, GCA_005280915.1, GCA_005281155.1, GCA_002208845.2, GCA_002903215.1, GCA_002919795.1, GCA_005281165.1, GCA_005281265.1, GCA_005281245.1, GCA_005281395.1, GCA_000238735.1, GCA_002843195.2, GCA_002042885.1, GCA_005280935.1, GCA_002903305.1, GCA_003255895.1, GCA_002946635.1, GCA_001317135.1, GCA_000521945.1, GCA_000158355.2, GCA_001281005.1, GCA_002918875.1, GCA_003019835.1, GCA_000783755.1, GCA_000521965.1, GCA_000388155.1, GCA_003195445.1, GCA_003070705.1, GCA_005281045.1, GCF_002864025.1, GCF_003175795.1, GCA_000734905.1, GCA_000759735.1, GCA_000582615.1, GCA_000312465.1, GCA_003015305.1, GCA_000982845.1, GCA_002919825.1, GCA_002871775.1, GCA_000937505.2, GCA_000342325.1, GCA_001546285.1, GCA_002918835.1, GCA_002918855.1, GCA_002934505.1, GCA_002918865.1, GCF_003114915.1, GCA_000937455.2, GCA_001022155.1, and GCA_001022275.1.

## Ethics Statement

The animal study was reviewed and approved by the Institutional Animal Care Committee at Nankai University.

## Author Contributions

BL and LJ conceived the project. CY and CQ purchased the strains. JW and SZ prepared the sample DNA for sequencing. CY and ZY conducted the comparative genomic analysis. CY, JW, and YW constructed the mutants and preformed the experiments. BL, LJ, and CY prepared the manuscript. All authors read and approved the final manuscript.

## Conflict of Interest

The authors declare that the research was conducted in the absence of any commercial or financial relationships that could be construed as a potential conflict of interest.

## Footnotes

1 http://www.ncbi.nlm.nih.gov/genome/annotation_prok

2 http://tree.bio.ed.ac.uk/software/figtree

3 https://www.kegg.jp/blastkoala/

4 http://mobyle.pasteur.fr/cgi-bin/portal.py#forms:txsscan

5 http://www.pathogenomics.sfu.ca/islandviewer/

6 http://sourceforge.net/projects/codonw

## Supplementary Material

FIGURE S1 Heatmap chart generated from distances calculated based on the ANI values of 129 *Citrobacter* strains. The colors in the heatmap represent pairwise average nucleotide identity (ANI) values, with a gradient from red (low) to yellow green (high).

TABLE S1 The list of strains in this research.

TABLE S2 The result of the Identification of Virulence Genes.

TABLE S3 The result of the Identification of Resistance Genes.

TABLE S4 The information of CARD datebase.

TABLE S5 The information of Group8-specific core genome.
